# A case report of renal oxalosis and secondary hyperoxaluria due to chronic high vitamin C consumption 

**DOI:** 10.5414/CNCS111462

**Published:** 2025-03-14

**Authors:** Ioannis Eleftherios Neofytou, Georgios Lioulios, Emmanouil Almaliotis, Dimitra Vasilia Daikidou, Aikaterini Mplatsa, Elias Minasidis

**Affiliations:** Nephrology Department, 424 General Military Hospital of Thessaloniki, Thessaloniki, Central Macedonia, Greece

**Keywords:** renal oxalosis, secondary hyperoxaluria, vitamin C, chronic kidney disease, calcium oxalate crystals

## Abstract

Renal oxalosis occurs from supersaturation of the urine with oxalate in the presence of calcium, resulting in deposition of calcium oxalate crystals within renal tissue and, consequently, progressive renal disease. One of the causes of secondary hyperoxaluria is a high intake of vitamin C, which exceeds the renal excretion capacity, and can induce renal oxalosis. We present a case involving a 67-year-old patient with chronic kidney disease and proteinuria, associated with secondary hyperoxaluria and renal oxalosis, who reported prolonged, excessive intake of vitamin C supplements. The patient presented with a gradual worsening of his renal function and proteinuria during the last 6-month period, after an episode of SARS-CoV-2 infection. The kidney biopsy revealed calcium oxalate crystals within the renal tissue. Thorough investigation and history-taking revealed a substantial increase in vitamin C supplementation during the SARS-CoV-2 infection (up to 3 g daily), indicating secondary hyperoxaluria as the causative factor. Overall during the pandemic, supplement consumption dramatically increased and patients were not adequately informed about the risks of various over-the-counter products. Excessive intake of vitamin C, popularized for its supposed health benefits, can lead, among others, to secondary hyperoxaluria and renal oxalosis. Prompt recognition is pivotal to initiate management and to prevent irreversible kidney damage.

## Introduction 

Renal oxalosis is a rare condition, characterized by the deposition of calcium oxalate crystals in the kidneys. Crystal deposition occurs due to hypersaturation of the urine with oxalate, which in the presence of calcium crystalizes primarily inside the tubules but this also can occur in the interstitium, leading to tubular injury, interstitial fibrosis, and ultimately progressive renal dysfunction. Apart from its genetic cause, primary hyperoxaluria, renal oxalosis is usually associated with secondary hyperoxaluria that arises mainly from increased intestinal absorption or excessive intake of oxalate precursors [1, 2]. Failure to promptly manage secondary hyperoxaluria can lead to serious renal damage and irreversible reduction of renal function [2, 3]. 

Vitamin C (ascorbic acid) is identified as a biologically essential micronutrient that plays a vital role in a variety of physiological processes. Common food sources of vitamin C include citrus fruits, berries, and green leafy vegetables [4]. Vitamin C plays a well-known role in collagen synthesis, immune function, iron absorption, and in particular acts as a potent antioxidant, protecting cells from harmful oxidative stress. However, high vitamin C intake increases the production of a potentially harmful byproduct, oxalate [3, 5]. 

Ascorbic acid is metabolized primarily in the liver, undergoing an initial non-enzymatic reversible oxidation to dehydroascorbic acid (DHA), while functioning as an antioxidant (by donating electrons) [6]. The enzyme dehydroascorbic acid reductase (DHAR), along with glutathione reductase, converts DHA back into ascorbic acid. If DHA is not reduced back to ascorbic acid, it undergoes irreversible hydrolysis to form 2,3-diketogulonic acid (2,3-DKG), a process assisted by the enzyme 2,3-diketogulonate decarboxylase. 2,3-DKG is non-enzymatically broken-down following either the L-xylosone and L-threonate pathway [7, 8, 9]. These non-toxic metabolites can either be excreted by the urine or further converted into various benign products. However, part of the L-xylosone pathway is a four-step process that includes two oxidizations, converting to glyoxal, glycolate, glycoxylate, and finally oxalate. This is assisted by lactate dehydrogenase (LDH) or glycolate oxidase (non-directly) [10, 11]. In healthy individuals, the amount of oxalate produced by vitamin C metabolism is marginal and does not pose a significant risk to normal kidneys. However, long-term, high-dose vitamin C supplementation (1 – 2 g daily is the upper limit for adults [12, 13]) can result in excessive oxalate concentrations, overwhelming the renal excretion capacity. This can lead to secondary hyperoxaluria and subsequent renal oxalosis [10, 14]. 

Healthcare providers should be well aware of the potential kidney complications that stem from excessive vitamin C intake. Prompt recognition of secondary hyperoxaluria as a causative factor of renal dysfunction is necessary to initiate an appropriate treatment plan and prevent further deterioration of renal function [15]. This comprehensive case report aims to elucidate the steps of clinical presentation, diagnostic evaluation, and treatment of a patient diagnosed with secondary hyperoxaluria and renal oxalosis following chronic high consumption of vitamin C. 

## Case report 

A 67-year-old Caucasian male patient presented to the nephrology department with concerns about deterioration in his laboratory values. He suffered from proteinuria of up to 3.2 g/d and gradual decline of renal function over the last 6 months (eGFR at presentation 12.5 mL/min/1.73m^2^). His medical history included chronic kidney disease, hypertension, dyslipidemia, hyperuricemia, carotid artery disease with stent placement 4 – 5 years prior, and an ischemic stroke with mild hemiplegic gait, which was confirmed during a previous hospital admission. Furthermore, 1 year before the current presentation, he had a history of moderate COVID-19 infection (mainly respiratory symptoms) that required hospitalization in a medical unit for COVID-19. His present medication included candesartan, felodipine, nebivolol, acetylsalicylic acid, clopidogrel, and a combination of ezetimibe/simvastatin. He has been a smoker for 30 years and in terms of professional history he is a retired firefighter. The hereditary history was unremarkable. 

The patient’s renal history started 15 years earlier, at the age of 52, when he was incidentally diagnosed with stage 3A chronic kidney disease during a pre-operative medical evaluation. At that time, he was found to have elevated creatinine levels (1.6 mg/dL, GFR: 55 mL/min/1.73m^2^) microscopic hematuria and proteinuria ~ 500 mg/day). Initially, the elevated creatinine levels were attributed presumably to poorly controlled hypertension; the patient was not monitored by a nephrologist, and no further investigation was performed during that period, despite medical advice. On presentation, the patient was afebrile, had a blood pressure of 135/75 mmHg and an ambient air saturation of 97%, and was in generally good condition. Physical examination revealed no notable findings other than a mild systolic murmur on mitral auscultation and gait abnormality consistent with his prior stroke. 

## Results 

Blood tests showed a gradual increase in creatinine levels over the past 6 months, rising from a baseline of 1.8 mg/dL to 2.5 mg/dL 3 months ago, 3.6 mg/dL 1 month ago, and currently 4.5 mg/dL. The eGFR had decreased to 12.5 mL/min, and proteinuria had reached 3.2 g per day. The rest of the laboratory testing revealed low hemoglobin (normochromic and normocytic) and mildly elevated parathormone levels (Table 1). Despite appropriate management of renal disease during hospitalization, including optimal blood pressure control and intravenous fluids to prevent volume contraction, creatinine levels remained relatively elevated. 

The patient was evaluated through screening for various systemic diseases, extensive laboratory blood tests, and imaging tests. The kidney disease progress could not be attributed to prerenal causes as the patient showed no sign of hypovolemia, no hypotensive episodes were documented or mentioned, the urea-to-creatinine ratio was not increased (< 40 : 1), and urinalysis results did not indicate concentrated urine (Table 1). 

Post-renal causes were also excluded. Computer tomography (CT) of the abdomen revealed no ureter dilation, kidney stones, or signs of retroperitoneal fibrosis. In addition, renal ultrasound showed bilaterally sized kidneys within the lower limits of normal (left 9.8 cm, right 10.2 cm). Prostate gland size was normal, with no evidence of marked hypertrophy, or post-void residual urine volume increase. Finally, no signs/symptoms of infection were observed or mentioned by the patient. 

Investigation of intrinsic renal etiologies yielded negative results as well. Blood gas analysis found normal acid-base balance, and electrolyte panels revealed no characteristic abnormalities suggestive of a tubular dysfunction. No evidence of autoimmune involvement was identified. The patient denied any symptoms typically associated with systemic autoimmune diseases, such as arthralgias or cutaneous manifestations. Additionally, laboratory tests for estimated sedimentation rate (ESR), complement levels, antineutrophil cytoplasmic antibodies (ANCA), antinuclear antibodies (ANA), and anti-double stranded DNA antibodies (anti-DNA) were negative. Serologic testing for common viral infections, including hepatitis B, hepatitis C, and HIV, was unremarkable. The assessment of hematological disorders was similarly negative. A complete blood count (CBC), peripheral blood smear microscopy, and serum and urine protein electrophoresis and immunofixation studies revealed no abnormalities. 

**Figure 1:**
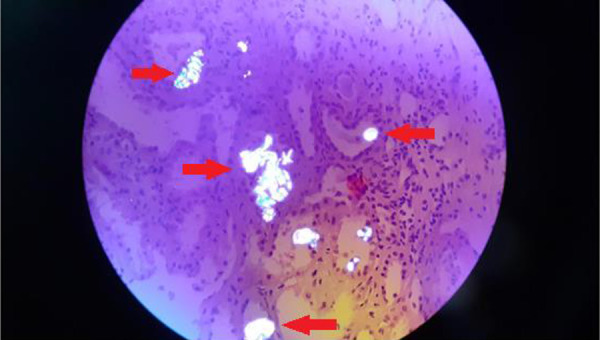
Kidney biopsy under polarized light: Oxalate crystal depositions seen in tubule lumens and interstitial spaces. Red arrows show the oxalate crystals. Magnification: × 400 (hematoxylin and eosin stain).

**Figure 2:**
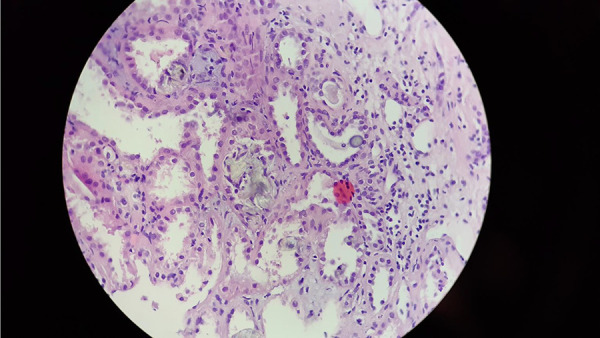
Prominent glomerulosclerosis (8 of 13 glomeruli examined), tubule atrophy, vasculopathy and interstitial fibrosis indicate advanced chronic kidney disease (CKD). Concurrent lesions of renal oxalosis and secondary focal segmental glomerulosclerosis (FSGS) strongly suggest the probable diagnosis. Magnification: × 400 H & E.

Taking into account the work-up discussed above, the following differential diagnoses were entertained: hypertensive nephropathy due to long-standing hypertension, nephropathy associated with the previous SARS-CoV-2 infection, and an unidentified progression of primary glomerulopathy. However, hypertensive nephropathy is more common in patients of African origin who carry the APOL1 gene variant, and this can increase the risk of hypertension-related end-stage renal disease by ~ 8 – 10 times. Therefore, hypertensive nephrosclerosis was a highly unlikely diagnosis to this patient. Additionally, the deterioration of renal function could not easily be attributed to a SARS-CoV-2 infection, as it appeared much later in the course of the infection than the onset of COVID-19 symptoms. Therefore, a kidney biopsy was performed (Table 2). The results reported extensive injuries in the renal tissue indicative of chronic kidney disease (glomerulosclerosis, tubular atrophy, vasculopathy). The immunofluorescence microscopy for IgG, IgA, IgM, C3, and C1q was negative, but the presence of clear crystals consistent with calcium oxalate deposition was found. Hematoxylin and eosin staining further confirmed the presence of crystals (Figures 1, Figure 2). 

In accordance with the biopsy findings, the patient was once more questioned in detail focusing on his diet (including a detailed daily dietary intake, specific herbs, spices, energy drinks, high-oxalate foods) and the consumption of over-the-counter products. After thorough history taking, no previous history of urolithiasis or hereditary kidney disease associated with oxalosis was reported. Finally, he revealed that he had been consuming oral vitamin C supplements in high daily doses for ~ 7 years. He reported a minimum daily intake of 1 g/d combined with an oxalate rich diet. Interestingly, during the SARS-CoV pandemic, and particularly when he experienced even mild respiratory infection symptoms, he increased the dose of vitamin C to 2 – 3 g/d. Therefore, secondary hyperoxaluria and renal oxalosis were attributed to the prolonged excessive vitamin C consumption. 

### Management and follow-up 

Following the diagnosis of secondary hyperoxaluria and renal oxalosis, a 24-hour urine collection revealed that urine citrate levels were significantly low (low 89 mg/24h, normal laboratory values: 434 – 1.191 mg/24h), and therefore failing to act proactively against crystal formation and disposition. In addition, the 24-hour urine oxalate measurement was performed and found to be moderately elevated at 45 mg/24h (normal range: 10 – 40 mg/24h), supporting the diagnosis. A less common cause of elevated urine oxalate is ethylene glycol consumption. The patient denied any intake of ethylene glycol or other toxic substances. However, a toxicology assessment was not performed because of laboratory constraints at the hospital [16]. At discharge, he was advised to completely avoid vitamin C supplements, to follow a poor in oxalate diet, and maintain a high fluid intake. Finally, he was prescribed supplements of HCO_3-_ and potassium citrate (2.7 g/d), and was recommended regular monitoring and treatment of hypertension, dyslipidemia, and hyperuricemia. However, due to economic constraints and limitations in insurance coverage, he could only afford the potassium citrate supplement. To manage his bicarbonate needs, he was advised to regularly consume half a teaspoon of baking soda dissolved in water. This alternative approach was chosen to maintain his bicarbonate levels more affordably. 

The patient was re-evaluated during the follow-up period, and the kidney function showed improvement. Three months after his hospitalization, the serum creatinine level was 2.9 mg/dL (eGFR 21.4 mL/min). He remained asymptomatic and had no signs of damage in other organs or systems due to the hyperoxaluria, as confirmed by cardiological and ophthalmological examination. 

## Discussion 

This case presentation aims to underscore hyperoxaluria as a potential cause of renal dysfunction and proteinuria. The development of secondary hyperoxaluria and renal oxalosis in this particular case is attributed to the patient’s excessive consumption of vitamin C over an extended period. It resulted in accumulation of calcium oxalate internally and deposition of crystals in the kidney, leading to progressive renal injury and deterioration of renal function [3, 17]. Due to the pre-existing stage 3A CKD, the kidneys’ reduced reserves were susceptible to the effects of oxalate formation and subsequent kidney damage caused by high vitamin C administration. As the glomerular filtration ability decreased, it led to an exacerbation of this condition as urinary oxalate excretion continued to decrease while its accumulation increased [18]. 

Beyond excessive vitamin C concentrations, other factors for secondary hyperoxaluria include increased intake of oxalate-rich foods (peanuts, rhubarb, spinach, beets, chocolate, and sweet potatoes), parenteral nutrition, fat malabsorption, inflammatory bowel diseases, intestinal bypass surgery, and certain medications or toxins (antiretrovirals and antibiotics, ethylene glycol) [19, 20, 21, 22]. Secondary hyperoxaluria can be a serious complication of kidney function but also of other organs, causing, for example, cardiomyopathy, anemia, and peripheral neuropathy [26]. A recent systematic metanalysis about secondary hyperoxaluria examined 10 case studies and 108 patients. Renal biopsies revealed that over 70% of patients appear to have serious tubular and interstitial damage. More than half of them eventually required renal replacement therapy [19]. 

Vitamin C is essential and must be obtained through exogenous sources as the body cannot produce it. The daily requirement for vitamin C varies among individuals. To maintain sufficient vitamin C levels, daily recommendations range from 40 to 120 mg, with pregnant women and smokers needing higher doses [23]. The minimum amount needed to prevent scurvy is 10 mg/d [24]. Individuals who consume a relatively balanced diet generally meet their daily vitamin C needs and do not require supplements [25]. It is important to note that at doses exceeding 100 mg, over 25% is excreted through the kidneys, and doses above 500 mg are almost entirely excreted, increasing the risk of stone formation in a dose-dependent manner [23, 26]. 

Many people worldwide opt for nutritional supplements, particularly vitamin C. Therefore, the benefits of vitamin C intake, especially in cancer, vascular health, and infections, have been significantly studied to determine its value [23]. Several clinical trials found no substantial advantage in cancer patients or for cancer prevention with high vitamin C doses [5, 27, 28]. Furthermore, vitamin C supplementation reduced the risk of coronary disease only in those with a vitamin C deficiency [29, 30]. The relationship between vitamin C and infections has been explored in numerous studies. However, a clear beneficial role for vitamin C has been observed only in animal studies, and clinical trial results remain inconclusive [31, 32, 33]. Consequently, vitamin C supplementation is not currently recommended in clinical guidelines except for preventing vitamin C deficiency and scurvy [34]. 

In the recent devastating pandemic crisis, various approaches have been tested for proactive protection against SARS-COV-2 infection. Vitamin supplements, especially those containing vitamin C or/and D, were suggested and even advertised as potential strategies for ameliorating disease severity and progress [35]. To date, there is no evidence that vitamin C supplementation has any clinical benefit on the course of COVID-19 infection [36]. During the pandemic, the demand for supplements increased significantly. For instance, only a few weeks after the spread of COVID-19, sales increased by ~ 45% in the United States compared to the previous year [37]. Consumers favored over-the-counter products primarily containing herbs such as vitamin C, vitamin D, zinc, selenium, turmeric, and garlic, presumably to bolster their immune systems [38]. 

People around the world continue to consume supplements in various forms, combinations and dosages [36]. While under normal conditions vitamin C supplementation is relatively harmless, the chronic high dose of vitamin C (especially above 1 g daily) in this patient led to a deterioration in his kidney function [39]. Unfortunately, patient awareness of the potential risks associated with administering over-the-counter products is often limited. In addition, it is common for patients to fail to report their consumption to healthcare professionals. Like other medications, over-the-counter products carry a significant risk of adverse events, including drug interactions, side effects, toxic overdose, reduced effectiveness of prescribed medications, and even death in severe cases [40]. 

The primary intent of this case report is to raise awareness of a lesser-known but likely consequence of administration of vitamin C in high doses, which physicians should take into consideration. We seek to alert and remind clinicians about the critical importance of meticulous history-taking, encompassing dietary habits and dietary supplement records, especially in cases of a diagnostic dilemma like this one [41]. In clinical practice, a number of confounding factors could mask the underlying reason behind a medical condition. In this particular patient, uncontrolled hypertension and recent COVID-19 infection initially prevented clinicians from exploring other possible causes of kidney disease progression [42, 43]. Nevertheless, identifying the root cause, discontinuing vitamin C consumption, and changing diet contributed significantly to managing this patient’s condition. 

In summary, this report is a valuable reminder of the risks associated with vitamin C. Healthcare professionals must exercise vigilance in taking thorough medical history, including recording dietary habits and the use of over-the counter products. It could further assist in accurately diagnosing medical conditions and individualizing patient management. 

## Conclusion 

Excessive vitamin C consumption can cause secondary hyperoxaluria and lead to the development of renal oxalosis and progressive kidney disease. Our comprehensive case report highlights the consideration of secondary hyperoxaluria in the differential diagnosis of renal dysfunction particularly when patients report high vitamin C intake for prolonged periods. Clinicians should be aware of the potential adverse events linked with excessive vitamin C intake, including renal oxalosis. Timely recognition of the disease, discontinuation of the causative factor, and treatment can improve kidney function while preventing complications associated with renal oxalosis. Renal function should be closely monitored over a long period of time and adherence to the treatment plan, including dietary and lifestyle changes, should be encouraged. 

## Availability of data and materials 

All data underlying the findings are fully available from the corresponding author upon request. 

## Ethics approval and consent to participate 

No Ethical Committee approval was required for this case report by the department because this article does not contain any studies with human participants or animals. Informed consent was obtained from the patient included in this study. 

## Consent for publication 

The patient gave his written consent to use his personal data for the publication of this case report and any accompanying images. 

## Authors’ contributions 

All authors contributed to study conception and design. Analysis, article structure, interpretation, manuscript drafting and revision were performed by IN. Acquisition and interpretation of data, manuscript drafting, and clinical patient care were performed by AE. Patient treatment, comments and revision of the text were performed by AM and DD. Overall supervising and critical revision for important intellectual content was performed by EM and GL. All the authors have read and approved the final version of the manuscript and agreed to be accountable for all aspects of the work. 

## Funding 

No funds, grants, or other support was received. 

## Conflict of interest 

The authors declare no conflict of interest and confirm accuracy. 

**Table 1.** Laboratory findings.

**Laboratory tests**	**Reference values**	**On presentation**	**On discharge**
Creatinine (mg/dL)	0.8 – 1.2	**4.5**	**3.37**
Urea (mg/dL)	5 – 20	**156.8**	**135.5**
Sodium (mg/dL)	137 – 145	**134**	**135**
Potassium (mg/dL)	3.5 – 5.2	**4.8**	**3.7**
Phosphate (mg/dL)	2.4 – 4.5	**4.1**	
Calcium (mg/dL)	8.4 – 10.2	**8.8**	**9**
Chloride (mEq/L)	95 – 105	**98**	
HCO_3-_ (mmol/L)	22 – 24	**18**	**23**
Hemoglobin (g/dL)	13.5 – 16	**12.5**	**10.5**
Mean corpuscular volume (fl)	80 – 95	**86**	**84.3**
White blood cells (×10^9^/L)	3,800 – 11,000	**8,820**	**11,100**
Platelets (per mL)	150 – 450,000	**205,000**	**155,000**
Ferritin (ng/mL)	12 – 300	**149,9**	
International normalized ratio	1 – 1.5	**1.01**	**1.02**
Aspartate aminotransferase (IU/L)	5 – 45	**24**	**40**
Alanine aminotransferase (IU/L)	8 – 55	**40**	**76**
Glucose (mg/dL)	75 – 99	**86.6**	**91**
Creatine phosphokinase (mcg/L)	20 – 120	**170**	**86**
Lactate dehydrogenase (IU/L)	140 – 280	**174**	**290**
Alkaline phosphatase (IU/L)	35 – 150	**38**	**68**
C-reactive protein (mg/dL)	0.04 – 0.5	**< 0.04**	**1.18**
Erythrocyte sedimentation rate (mm/h)	2 – 15	**16**	
Parathormone (pg/mL)	10 – 65	**127.3**	
Total protein (g/dL)	5.5 – 8	**6.1**	**6.9**
Albumin (g/dL)	3.4 – 5.3	**4.5**	**4.8**
Uric acid (mg/dL)	3.5 – 7	**9**	
Total bilirubin (mg/dL)	0.1 – 1.2	**0.5**	
Triglycerides (mg/dL)	90 – 150	**117.8**	
Complement C4 (mg/dL)	15 – 45	**22.3**	
Complement C3 (mg/dL)	75 – 175	**115.6**	
ANA (Antinuclear Antibodies)	< 1:40	**Negative**	
Anti – DNA (IU/mL)	< 10	**Negative**	
P – ANCA (IU/mL)	< 1.4	**Negative**	
C – ANCA (IU/mL)	< 2.8	**Negative**	
Prostate specific antigen (ng/mL)	0.6 – 4.0	**0.94**	
HIV Ag/Ab	< 0.9	**0.05**	
HCV antibodies (log IU/mL)	< 0.8	**0.11**	
Australian antigen- HBsAg (IU/mL)	< 0.5	**0.27**	
Urinalysis			
Appearance	Clear	Light yellow	
Specific gravity	1,005 – 1,030	**1,012**	
pH	4.5 – 8	**5**	
Albumin	Negative	**+/++**	
Hemoglobin	Negative	**++**	
Glucose	Negative	**–**	
Urocholine	Negative	**–**	
Nitrites	Negative	**–**	
Leukocytes	Negative	1 – 2 HPF	
Erythrocytes	Negative	1 – 2 HPF	
Squamous epithelia	Negative	–	
Mucus	Negative	Positive	
Microorganisms	Negative	–	
Crystals	Negative	–	
Pathological casts	Negative	–	

HPF = high-power field. Bold = patient values.

**Table 2.** Kidney biopsy report.

**Macroscopic findings of tissue**
3 Cylinders: length 5 – 10 mm for histochemistry 1 Cylinder: length 3 mm, freshly frozen for immunofluorescence/ immunohistochemistry
**Microscopic findings**
**1. Histochemistry** 13 glomeruli from the renal cortex
**Glomeruli** 8 of them, globally or almost globally sclerosed 5 of them, show in parts mild thickness increase of mesangium, one of them segmentally sclerotic, staining with Congo red: negative for amyloidosis
**Juxtaglomerular apparatus ** No remarkable changes
**Tubules** In several places, acute tubular damage with flattening of tubular epithelial cells. Some tubules contain PAS (+) casts. Calcium oxalate crystals are found in several tubules. In ~ 50% of the cortex there is tubular atrophy.
**Interstitial tissue ** Interstitial fibrosis: ~ 50% of the area, combined with chronic inflammatory infiltration
**Vessels** Arterioles: mild/ moderate hyalinosis Arteries: extensive thickening of the intima/ media tunica Veins: without injury
**2. Immunofluorescence (0 – 3+) ** Specimen not in condition to be evaluated
**3. Ιmmunohistochemistry ** No specific positivity: IgG, IgA, IgM, C3, and C1q
**Conclusion**
Percentage of globally sclerosed glomeruli: 61.6% (8/13) Percentage of ischemic glomeruli: 0% Percentage of cortex with tubular atrophy – interstitial fibrosis: 50% Arterioles: mild/ moderate hyalinosis Arteries: Extensive thickening of intima/media tunica Extensive injuries in the renal tissue indicative of chronic kidney disease (total glomerulosclerosis, tubular atrophy, vasculopathy), findings of renal oxalosis and secondary focal segmental glomerulosclerosis.

PAS = Periodic acid-Schiff stain.
